# Schlafen family is a prognostic biomarker and corresponds with immune infiltration in gastric cancer

**DOI:** 10.3389/fimmu.2022.922138

**Published:** 2022-08-25

**Authors:** Jiannan Xu, Songyao Chen, Jianming Liang, Tengfei Hao, Huabin Wang, Guangyao Liu, Xinghan Jin, Huan Li, Junchang Zhang, Changhua Zhang, Yulong He

**Affiliations:** ^1^ Digestive Diseases Center, The Seventh Affiliated Hospital of Sun Yat-sen University, Shenzhen, China; ^2^ Department of Thoracic Surgery, The Third Affiliated Hospital of Sun Yat-sen University, Guangzhou, China; ^3^ Division of Hematology/Oncology, Department of Pediatrics, The Seventh Affiliated Hospital of Sun Yat-sen University, Shenzhen, China; ^4^ Center of Gastrointestinal Surgery, The First Affiliated Hospital of Sun Yat-sen University, Guangzhou, China

**Keywords:** SLFN family, Schlafen, prognostic biomarker, gastric cancer, tumor immune cell infiltration

## Abstract

The Schlafen (SLFN) gene family plays an important role in immune cell differentiation and immune regulation. Previous studies have found that the increased SLFN5 expression in patients with intestinal metaplasia correlates with gastric cancer (GC) progression. However, no investigation has been conducted on the SLFN family in GC. Therefore, we systematically explore the expression and prognostic value of SLFN family members in patients with GC, elucidating their possible biological function and its correlation with tumor immune cells infiltration. TCGA database results indicated that the SLFN5, SLFN11, SLFN12, SLFN12L, and SLFN13 expression was significantly higher in GC. The UALCAN and KM plotter databases indicated that enhanced the SLFN family expression was associated with lymph node metastasis, tumor stage, and tumor grade and predicted an adverse prognosis. cBioportal database revealed that the SLFN family had a high frequency of genetic alterations in GC (about 12%), including mutations and amplification. The GeneMANIA and STRING databases identified 20 interacting genes and 16 interacting proteins that act as potential targets of the SLFN family. SLFN5, SLFN11, SLFN12, SLFN12L, and SLFN14 may be implicated in the immunological response, according to Gene Set Enrichment Analysis (GSEA) and Kyoto Encyclopedia of Genes and Genomes (KEGG). Additionally, Timer and TISIDB databases indicate that SLFN5, SLFN11, SLFN12, SLFN12L, and SLFN14 are involved in the immune response. Furthermore, Timer, TCGA, and TISIDB databases suggested that the SLFN5, SLFN11, SLFN12, SLFN12L, and SLFN14 expression in GC is highly linked with immune cell infiltration levels, immune checkpoint, and the many immune cell marker sets expression. We isolated three samples of peripheral blood mononuclear cell (PBMC) and activated T cells; the results showed the expression of SLFN family members decreased significantly when T cell active. In conclusion, the SLFN family of proteins may act as a prognostic indicator of GC and is associated with immune cell infiltration and immune checkpoint expression in GC. Additionally, it may be involved in tumor immune evasion by regulating T cell activation.

## Introduction

Gastric cancer (GC) has one of the highest morbidity and mortality rates globally (1). According to the 2020 global cancer statistics, there are 1.09 million new GC cases worldwide and ranked fifth in the world’s new cancer incidence rate (2). Additionally, there were 770,000 deaths, accounting for one-fourth of all cancer deaths (2).

GC grows slowly and asymptomatically in the early stages, making it difficult to detect (3). Most patients were diagnosed in the middle or late stages, when the tumor had invaded surrounding tissue or distant metastasis has occurred, due to GC’s hidden symptoms and the low popularity of early cancer screening (4). The patient’s survival rate with GC has improved significantly due to early detection, treatment, and the availability of multiple treatment options. Nonetheless, the average five-year survival rate of GC patients in most countries remains less than 40% (1). Elucidating GC development and progression mechanism is important in devising novel treatment targets and designing new drugs against new targets to increase GC patient’s survival.

The positive rate of PD-L1 expression in GC ranges from 12–50%, which is closely related to tumor-infiltrating immune cells, particularly CD8^+^ T cells (5). According to studies, immune microenvironment characterization in tumors can be utilized as a diagnostic factor for immunotherapy efficacy, and PD-L1 expression in tumor-infiltrating immune cells is more beneficial for predicting effective treatment (6). Therefore, new markers that detect tumor-associated infiltrating immune cells and new immunological checkpoints have high therapeutic relevance for GC immunotherapy.

Schwarz discovered the SLFN family in mice in 1988, and SLFN1 was the first identified member (7). This protein was named after the German word “Schlafen,” which means “sleep;” because of its capacity to maintain T cell quiescence by inducing T cell cycle arrest (7). SLFN family has many members, and humans have six SLFN: SLFN5, SLFN11, SLFN12, SLFN12L, SLFN13, and SLFN14 (8). In recent years, the SLFN family has been found to play an important role in tumor development and drug resistance (9). High SLFN5 expression in melanoma (10), renal cell carcinoma (11), and breast cancer (12) can inhibit tumor invasion and migration, indicating that SLFN5 acts as a tumor suppressor gene. However, high SLFN5 expression in glioblastoma (13), pancreatic ductal carcinoma (14), and prostate cancer (15) can promote tumor proliferation, invasion, and metastasis. Furthermore, studies have demonstrated that SLFN11 is an important chemotherapy-sensitizing gene in tumors. The high SLFN11 expression in colorectal cancer and lung adenocarcinoma causes tumor sensitivity to irinotecan chemotherapy and platinum chemotherapy drugs (16–18). Combining SLFN12 with phosphodiesterase 3A (PDE3A) can enhance the lung adenocarcinoma cells sensitivity to DNMDP (19).

In GC, SLFN4 is a biomarker of intestinal metaplasia (precancerous lesions of GC) in mice (20, 21). When mice are infected with *H. Pylori*, SLFN4+ cells migrate to the stomach and express the marker of bone marrow-derived inhibitory cells (MDSC), inhibiting T cell proliferation (20, 21). Similarly, SLFN12L (human homologue of 4) colocalized with cells expressing the MDSC surface markers, i.e., CD15^+^CD33^+^HLA-DR^lo^ (22). Therefore, the study suggests that human SLFN, such as SLFN12L (a homologue of SLFN4), may serve as a biomarker for detecting human gastric mucosal pre-tumor transformation. Furthermore, studies have demonstrated that higher SLFN5 expression in patients with intestinal metaplasia of gastric mucosa cells is connected to the GC progression (23). The authors also found that SLFN5 co-localize with T cells and M2-type macrophages, suggesting that SLFN5 plays an immunosuppressive role in GC (23).

The SLFN family has not yet been explored in GC and this study aims to systematic explore the expression and prognostic value of SLFN family members and determine their potential molecular function and correlation with tumor immune cell infiltration in GC.

## Materials and methods

### The SLFN family mRNA expression

The SLFN family mRNA expression data were collected from The Cancer Genome Atlas (TCGA database, https://www.cancer.gov/tcga). The database involved more than 20,000 molecularly characterized primary cancer, and matched normal samples spanning 33 cancer types. We analyzed the SLFN family’s RNA expression in 33 tumors using TCGA database. Moreover, SLFN family mRNA expression in GC tissues and adjacent normal tissues was compared using paired and unpaired tests. We used UALCAN database (http://ualcan.path.uab.edu/) to explore the clinical pathology parameters (tumor grade, lymph node metastasis status, and tumor stage) associated with SLFN mRNA expression in GC. UALCAN is a comprehensive web resource for cancer OMICS data analysis and gene expression data, especially the associated of gene expression level to clinical characteristics (24).

### SLFN family genome changes

The genetic alterations (types and frequency genomic changes) of the SLFN family in GC were investigated using the cBioPortal database (http://cbioportal.org). The cBioPortal database contains large-scale cancer genomic datasets that can be visualized, downloaded, and analyzed (25).

### Gene-gene interaction and protein-protein interaction networks

Gene networks, including SLFN family genes, were constructed using the GeneMANIA database (http://www.genemania.org). GeneMANIA database is a flexible, fast web interface used to generate gene-gene network and predict gene function (26).The proteins interacting with SLFN family members were identified using the STRING database (https://string-preview.org/), which is a database of known and predicted protein-protein interactions, and a protein network interacting with SLFN was built.

### Functional enrichment analysis

TCGA database was utilized to investigate the genes related to the SLFN family, and most relevant genes were selected for KEGG enrichment analysis using David database. LinkedOmics database, (http://www.linkedomics.org/login.php) which is a unique platform used to analyze cancer multi-omics data of cancers, was used to conduct gene set enrichment analyses (GSEA) on each SLFN member, including biological process (BP) and KEGG. The database can perform enrichment analysis based on Gene Ontology, biological pathways, network modules, etc (27).

### Survival analysis

KM Plotter database (http://kmplot.com), an online database use to investigate the correlation between gene expression and survival in patients with cancer (28), was utilized to study the association between SLFN family expression and GC patient survival time (OS, PFS) and to assess the prognostic value of SLFN family expression in GC. We assessed overall survival (OS) and progression-free survival (PFS). The TCGA database was used to study the genes not included in KM Plotter. Additionally, we used the KM Plotter database to examine the link between SLFN members associated with the prognosis of GC patients (*p* < 0.05) and clinical characteristics such as T stage, N stage, M stage, gender, age, and chemotherapy.

### Tumor-infiltrating immune cells

TIMER database (https://cistrome.shinyapps.io/timer/), a web resource used to evaluate different immune cells infiltration in diverse cancer types (29), was used to explore the link between tumor immune cell infiltration and SLFN family expression including B cells, CD8^+^ T cells, CD4^+^ T cells, neutrophils, macrophages, and dendritic cells. Tumor immune cell infiltration was further validated using the TISIDB (http://cis.hku.hk/TISIDB/index.php). TISIDB is another web portal for investigating the interaction of tumor and immune system (30). Meanwhile, the correlation module of the GEPIA database (http://gepia.cancer-pku.cn/index.html), which provides gene expression and correlation analysis in tumor/normal tissue (31), was utilized to investigate the association between SLFN expression and different gene marker sets of immune cells. The correlation and statistical significance of SLFN expression and immune infiltration were determined.

### Immune checkpoint analysis

The immunomodulator module of the TISIDB database (http://cis.hku.hk/TISIDB/index.php) was used to analyze the correlation between SLFN family expression and immune checkpoints, including CD160, CD244, CD247, CTLA4, LAG3, PDCD1, PDCD1LG2, TIGIT, and HAVCR2. As SLFN12L and SLFN14 were excluded in the TISIDB database, GEPIA database was used to analyze their correlation.

### Peripheral blood mononuclear cell and the correlation analysis

We analyzed SLFN family expression in the peripheral blood monocyte lineage using the immune cell module of the Human Protein Atlas database (https://www.proteinatlas.org/), which showing the expression of genes in immune cell types (32), and then isolated PBMC from healthy individuals. After 24 h, CD3/CD28 were added to activate T cells and extract mRNA. Then, qPCR was performed to analyze the changes in SLFN mRNA expression before and after T cell activation.

### PBMC extraction and culture

Three blood samples were collected from patients admitted to the Digestive Medicine Center of the Seventh Affiliated Hospital of Sun Yat-sen University. Before blood extraction, patients were informed of the purpose and the consent forms were signed. The study was approved by the Ethics Committee of the Seventh Affiliated Hospital of Sun Yat-sen University (Ethics number: KY-2021-118-01). The isolated PBMC were cultivated in X-Vivo15 lymphocyte culture media with a 1:1000 ratio of IL-2 and 1:1000 ratio CD3/CD28, then placed in a cell culture box containing 5% CO_2_ at 37°C. The medium was changed or subcultured according to the cell growth conditions.

### Quantitative real-time PCR

Total RNA was extracted from PBMC and GC tissue using Trizol reagent. The RNA yield and purity were determined using NanoDrop spectrophotometer (NanoDrop Technologies). RNA was reverse transcribed to make cDNA using cDNA synthesis kit (Accurate Biology). The reverse transcription-PCR was performed on a BioRAD Real-Time PCR System, and the gene expression was normalized using GAPDH primers. The primers for qRT-PCR are shown in **Table S1**.

### Statistical analysis

GraphPad Prism 5.0 (California) was used for data statistics and plotting the results. Chi-square and rank-sum tests were used for classification and count variables. T test was used for comparison of two groups. Comparisons among multiple groups involved one-way ANOVA and Bonferroni test was used for correction p-values. Generally, *p*-values less than 0.05 or less than correction *p* were considered statistically significant. This indicates that *p* > 0.05, *p < 0.05, **p < 0.01, and ***p < 0.001.

## Results

### Increased SLFN family expression in GC

**Figure 1:**
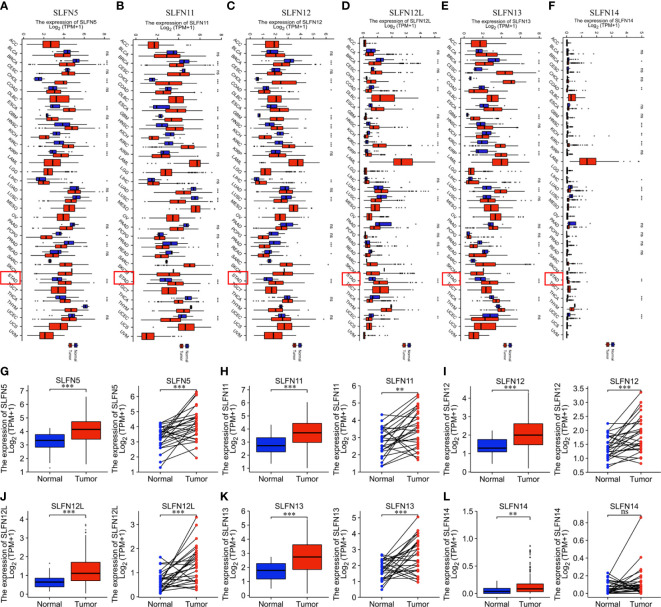
SLFN family members are highly expressed in gastric cancer. **(A–F)** Increased expression of SLFN family members in pan-cancer. **(A)** mRNA expression of SLFN5 in different tumors. **(B)** mRNA expression of SLFN11 in different tumors. **(C)** mRNA expression of SLFN12 in different tumors. **(D)** mRNA expression of SLFN12L in pan-cancer. **(E)** mRNA expression of SLFN13 in pan-cancer. **(F)** mRNA expression of SLFN5 in pan-cancer. **(G–L)** Paired and unpaired mRNA expressions of different SLFN family members in gastric cancer tissues and normal gastric mucosa tissues. **(G-K)** The paired and unpaired expressions of SLFN5, SLFN11, SLFN12, SLFN12L, and SLFN13 in tumors were higher than normal. **(L)** SLFN14 expression was higher in tumors than in normal tissues, but there was no difference in SLFN14 expression in paired tissues. *p < 0.05, **p < 0.01, ***p < 0.001 and ****p < 0.0001 and ns means no significance.

We used the TCGA database to investigate the SLFN mRNA expression between tumors and normal tissues in different tumors. The findings revealed that SLFN5, SLFN11, SLFN12, SLFN12L, SLFN13, and SLFN14 were expressed at higher levels in many cancers, including GC (stomach adenocarcinoma; STAD), while the SLFN14 expression levels were relatively low in all tumor and normal tissues (**Figures 1A–F**). We analyzed SLFN expression in paired and unpaired tumor tissues and normal gastric tissue using the TCGA database to investigate further the expression and clinical significance of the SLFN family in GC. The results demonstrated that SLFN5, SLFN11, SLFN12, SLFN12L, and SLFN13 expression in tumor tissues was significantly higher than that in normal tissues in paired, and unpaired tests, implying that they may play a role in promoting tumor progression in GC. While the SLFN14 expression levels in paired tissues were not statistically significant (**Figures 1G–L**).

**Figure 2:**
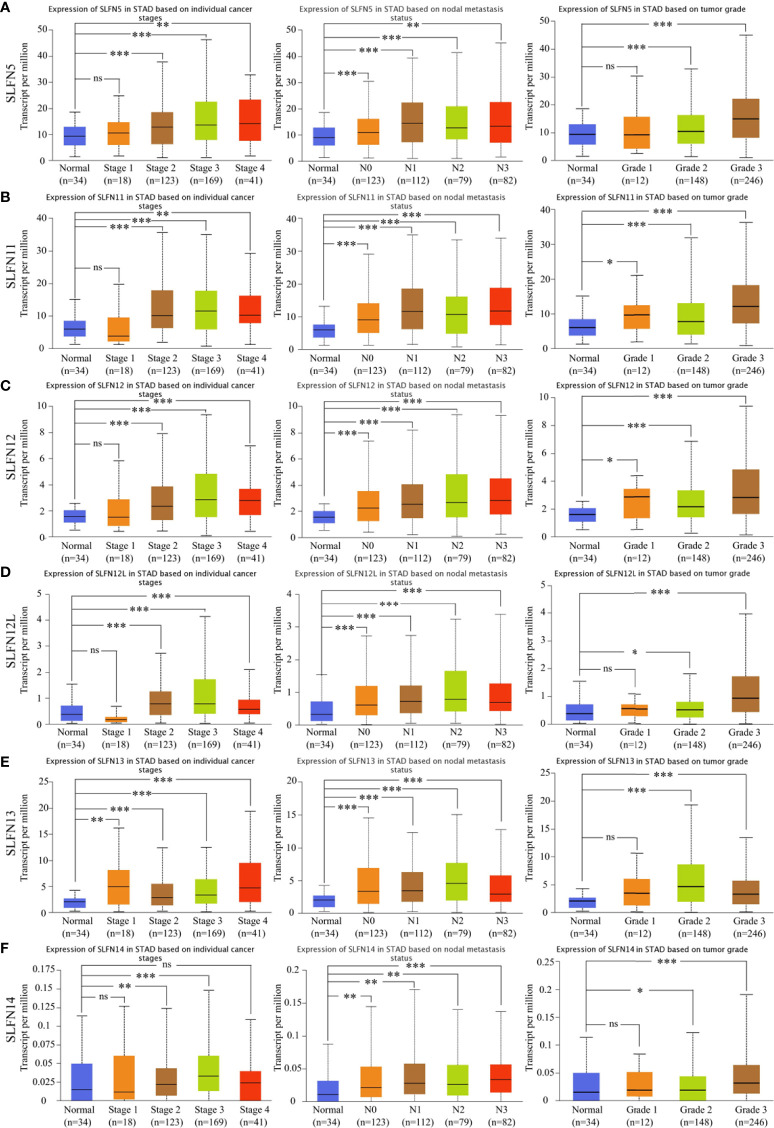
High expression of SLFN family members was correlated with gastric cancer stage, grade, and lymph node metastasis. **(A-F)** The mRNA expressions of SLFN5, SLFN11, SLFN12, SLFN12L, and SLFN13 were positively correlated with gastric cancer stage, tumor grade, and lymph node metastasis. *p < 0.05, **p < 0.01, and ***p < 0.001 and ns means no significance.

Following that, we investigated the roles of SLFN5, SLFN11, SLFN12, SLFN12L, and SLFN13 in GC. UALCAN database was utilized to explore the relationship between the expression and tumor stage, lymph node metastasis, and tumor grade. The results showed that the high SLFN5, SLFN11, SLFN12, SLFN12L, and SLFN13 expression in GC was positively correlated with lymph node metastasis, tumor stage, and tumor grade (**Figures 2A–F**). The findings further suggest that SLFN family may play a role in GC progression.

### Prognostic value of SLFN family in GC

**Figure 3:**
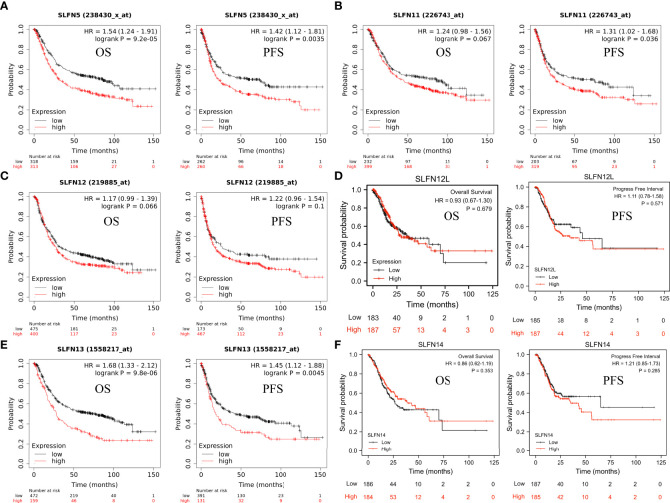
Prognostic value of SLFN family in GC. **(A-F)** The relationship between mRNA expression of different SLFN family members and OS and PFS in gastric cancer. **(A, E)** High SLFN5 and SLFN13 mRNA expression associated with poor OS and PFS in patients with gastric cancer.

Next, we investigated whether the SLFN family expression is associated with a poor prognosis in GC. The Kaplan-Meier plotter was used to analyze the OS and PFS of six SLFN GC members. The results showed that high SLFN5 and SLFN13 expression indicate poor prognosis in GC patients. OS [HR: 1.54 (1.24–1.91), *p* = 9.2×10^-5^; HR: 1.68 (1.33–2.12), *p* = 9.8×10^-6^, respectively] and PFS [HR: 1.42 (1.12–1.81), *p* = 0.0035; HR: 1.45 (1.12–1.88), *p* = 0.0045, respectively] (**Figures 3A, E**). As for SLFN11, SLFN12, SLFN12L and SLFN14, the OS have no statistically significant differences between the high expression group and low expression group in patients with GC (p>0.05, **Figures 3B–D, F**). These findings suggest that SLFN5 and SLFN13 promote GC progression and act as a prognostic indicator. We further analyzed the effects of SLFN5 and SLFN13 mRNA expression on prognostic association with clinicopathological characteristics of GC patients. The result revealed that high SLFN5 expression was associated with a poor prognosis in patients with clinicopathological characteristics, including male, any T stage, N0 and N+ stage, M0 stage, moderately differentiated, surgically treated, and HER2-positive. While SLFN13 overexpression in males and females, any T stage, N+ stage, M0 stage, moderately differentiated, surgically treated, and HER2-positive patients had a poor prognosis (**Tables 1**, **2**).

**Table 1:** Correlation of SLFN5 mRNA expression and clinical prognosis in GC with different clinicopathological factors by Kaplan-Meier plotter.

Clinicopathological characteristics	OS(N=631)				PFS(N=522)			
	N	Hazard ratio		p value	N	Hazard ratio		p value
Sex								
Female	187	1.46(0.95-2.24)		0.081	179	1.28(0.84-1.97)		0.25
Male	349	1.66(1.24-2.23)		0.00061a	341	1.58(1.18-2.1)		0.0019a
T Stage								
1	–	–		–	–	–		–
2	241	1.67(1.09-2.55)		0.0017^a^	239	1.37(0.91-2.07)		0.13
3	204	1.41(1-2)		0.05^a^	204	1.34(0.96-1.88)		0.084
4	38	2.48(1.06-5.82)		0.031^a^	39	3.94(1.77-8.79)		0.00034^a^
N Stage								
N0	74	3.65(1.23-10.8)		0.013^a^	–	–		–
N+	422	1.59(1.22-2.07)		0.00047^a^	423	1.42(1.1-1.83)		0.0065^a^
M Stage								
M0	444	1.54(1.17-2.04)		0.0021^a^	443	1.41(1.08-1.84)		0.011^a^
M1	56	1.57(0.79-3.1)		0.19	56	0.62(0.34-1.11)		0.1
Differentiation								
Poorly	121	0.51(0.29-0.87)		0.013 ^a^	121	0.52(0.32-0.85)		0.0082 ^a^
Moderately	67	1.78(0.85-3.71)		0.12	67	1.74(0.86-3.52)		0.12
Well	–	–		–	–	–		–
Treatment								
Surgery	380	1.41(1.06-1.88)		0.019 ^a^	375	1.28(0.97-1.69)		0.083 ^a^
5-FU based adjuvant	34	0.51(0.17-1.52)		0.22	34	0.48(0.19-1.24)		0.12
others adjuvant	76	4.09(1.48-11.29)		0.0032 ^a^	80	3.15(1.37-7.34)		0.005 ^a^
HER2								
Negative	429	1.3(1-1.69)		0.053	356	0.77(0.56-1.05)		0.099
Positive	202	2.25(1.54-3.29)		1.8*10^-5 ^a^	166	2.45(1.6-3.76)		2.4*10^-5 ^a^

a means p<0.05.

**Table 2:** Correlation of SLFN13 mRNA expression and clinical prognosis in GC with different clinicopathological factors by Kaplan-Meier plotter.

Clinicopathological characteristics	OS(N=631)					PFS(N=522)					
	N	Hazard ratio		p value		N		Hazard ratio		p value	
Sex											
Female	187	1.36(0.85-2.15)		0.19		179	1.35(0.86-2.13)			0.19	
Male	349	1.66(1.21-2.26)		0.0014 a		341	1.35(1.01-1.81)			0.043 a	
T Stage											
1	–	–		–		–	–			–	
2	241	1.36(0.87-2.13)		0.17		239	0.73(0.48-1.12)			0.15	
3	204	1.69(1.14-2.5)		0.0083 a		204	1.64(1.12-2.4)			0.0094 a	
4	38	0.34(0.14-0.84)		0.015 a		39	0.36(0.16-0.81)			0.011 a	
N Stage											
N0	74	0.58(0.25-1.34)		0.2		–	–			–	
N+	422	1.54(1.16-2.05)		0.0029 ^a^		423	1.38(1.05-1.82)			0.02 ^a^	
M Stage											
M0	444	1.49(1.12-1.98)		0.006 ^a^		443	1.4(1.06-1.83)			0.016 ^a^	
M1	56	0.71(0.39-1.3)		0.26		56	0.51(0.28-0.94)			0.029 ^a^	
Differentiation											
Poorly	121	0.54(0.32-0.89)		0.014 ^a^		121	0.55(0.34-0.89)			0.0014 ^a^	
Moderately	67	2.07(1.07-4)		0.027		67	1.83(0.97-3.46)			0.06	
Well	–	–		–		–	–			–	
Treatment											
Surgery	380	1.32(0.98-1.77)			0.064	375	1.23(0.93-1.63)			0.15	
5-FU based adjuvant	34	0.63(0.24-1.63)			0.35	34	0.49(0.21-1.18)			0.11	
others adjuvant	76	0.55(0.22-1.39)			0.2	80	1.5(0.65-3.48)			0.34	
HER2											
Negative	429	1.36(1.02-1.82)			0.035 ^a^	356	1.24(0.88-1.75)			0.23	
Positive	202	2.18(1.49-3.17)			3*10^-5	166	2.65(1.74-4.04)			2.7*10^-6 ^a^	

a means p<0.05.

### Genomic alterations of the SLFN family and the interaction network

**Figure 4:**
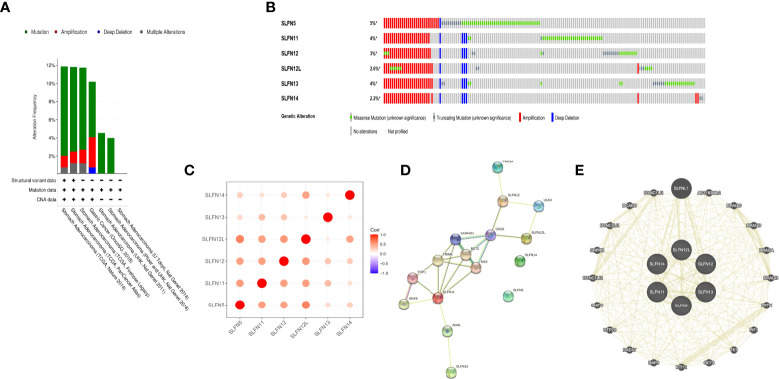
Genome changes and interaction network of SLFN family. **(A, B)** Genomic changes of SLFN gene families. **(C)** Correlation analysis among SLFN family members. **(D)** STRING generates PPI network of SLFN gene family. **(E)** GeneMania was used to construct SLFN family gene-gene interaction network.

The amplification frequency and mutation type of the SLFN family in GC were determined using the cBioportal database. The findings revealed that the SLFN family had a high frequency of genetic alterations in GC (about 12%), including mutations and amplification. SLFN5 gene alterations were the highest among the family, accounting for around 5% (**Figures 4A, B**). Additionally, we analyzed the correlation of six SLFN members family and found that they were highly correlated (**Figure 4C**). The gene network with probable interactions with the SLFN family and the protein-protein interaction network was then plotted using GeneMANIA and STRING databases. We identified 20 interacting genes and 16 interacting proteins that act as a potential target of SLFN family, as depicts in **Figures 4D, E**.

### Potential functions of the SLFN family in GC

**Figure 5:**
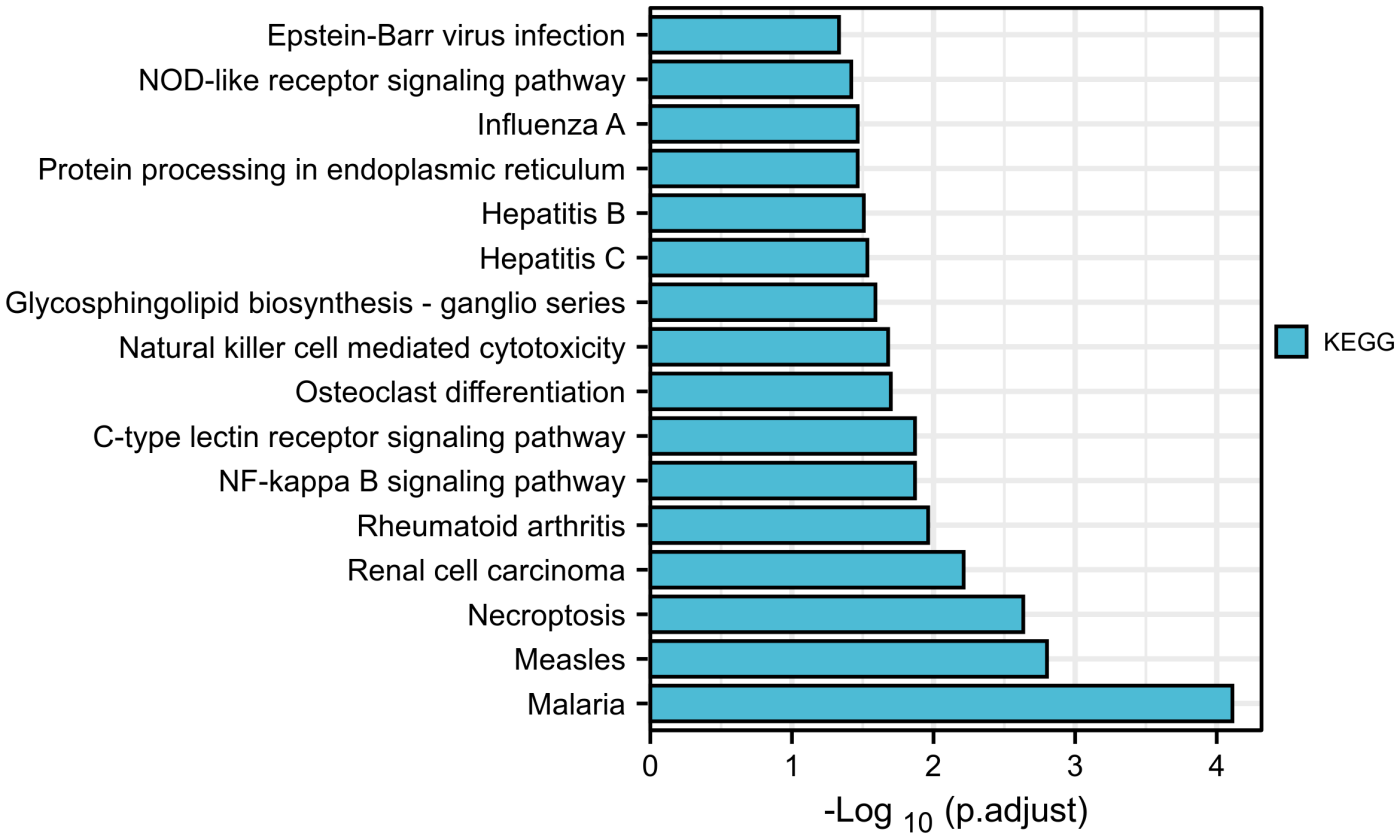
KEGG enrichment analysis of SLFN gene family. KEGG enrichment analysis suggested that SLFN family was associated with infectious and inflammatory diseases, including malaria, measles, rheumatoid arthritis, Epstein-Barr virus infection, hepatitis B, hepatitis C, etc. The NF-kB signal pathway are related.

**Figure 6:**
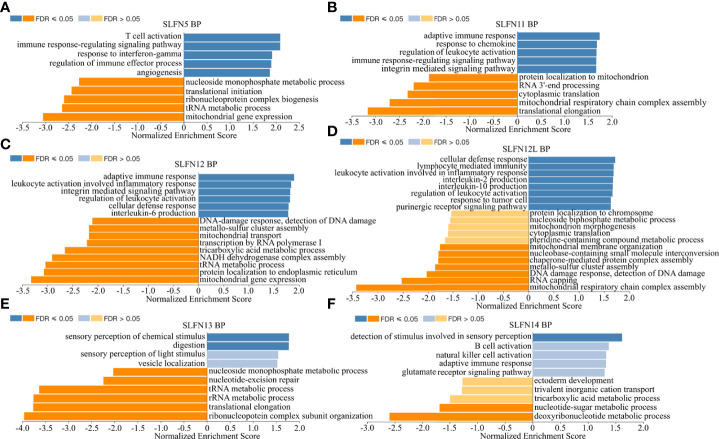
Biological functions of SLFN family members. Using Gene Set Enrichment Analysis (GSEA) module of LinkedOmics database to conduct functional enrichment for different SLFN members. **(A)** The biological process of SLFN5 suggests related to T cell activation, immune response, and immune regulation. **(B)** The biological process of SLFN11 related to adaptive immune response and immune regulation. **(C)** The biological process of SLFN12 related to adaptive immune response, leukocyte activation. **(D)** The biological function of SLFN12L related to lymphocyte-mediated immune response and inflammatory mediated leukocyte activation. **(E)** The biological function of SLFN13 is related to stimulus-response. **(F)** The biological function of SLFN14 is related to B cell activation.

There have been few studies on the function of SLFN family in GC, and our prior research found that SLFN family, especially SLFN5 and SLFN13, may promote GC. We performed a KEGG gene set enrichment analysis of the SLFN family to better understand its role in GC. The results revealed that the family was primarily associated with infectious and inflammatory diseases, such as malaria, measles, EB virus infection, rheumatoid arthritis, hepatitis B and hepatitis C. Furthermore, SLFN family is linked to NF-kappa B signaling pathways (**Figure 5**). We used GSEA module of LinkedOmics database to analyze BP and KEGG of each member to better understand the role of each family member in GC. The findings revealed that the functions of SLFN members were mainly related to immunity. Specifically, SLFN5 is associated with T cell activation and immune response regulation, SLFN11 with adaptive immune response and immune regulation, SLFN12 with adaptive immune response and leukocyte activation involved in inflammatory response and SLFN12L is related to lymphocyte-mediated immunity response and leukocyte activation involved in inflammatory response. SLFN13 is associated with stimulus-response, whereas SLFN14 is associated with B cell activation (**Figures 6A–F** and **Figure S1**).

### SLFN family correlation with immune cell infiltration in GC

**Figure 7:**
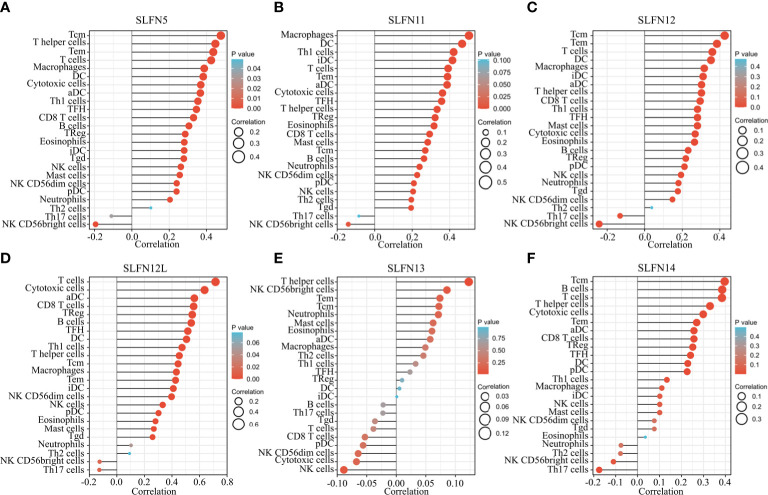
Relationship between SLFN family members and tumor immune cell infiltration. **(A–F)** The relationship between SLFN family members and immune cell infiltration in gastric cancer was analyzed using TCGA database. **(A, C, D)** SLFN5, SLFN12, and SLFN12L were mainly related to T cell infiltration, especially Tcm cell infiltration. **(B)** SLFN11 was mainly associated with macrophages and DC cells. **(E)** SLFN13 had no significant relationship with tumor immune cell infiltration. **(F)** SLFN14 was associated with Tcm and B cell infiltration.

Our previous research indicates that the SLFN family is involved in GC and is functionally related to inflammatory disorders, immune cell activation, and regulation. To determine whether the SLFN family plays a role in the immune microenvironment of GC, we used TIMER and TCGA databases to examine the relationship between SLFN family member expression and tumor immune cell infiltration, including B cells, CD4^+^ T cells, CD8^+^ T cells, neutrophils, macrophages, and dendritic cells. The findings revealed that SLFN5, SLFN11, SLFN12, and SLFN12L expression in GC significantly positively correlated with the infiltration of CD8^+^ T and CD4^+^ T cells, macrophages, neutrophils, and dendritic cells, but not with the infiltration of B cells SLFN14 were significantly positively correlated with all of these immune cells, including B cell. Additionally, SLFN13 expression was not significantly associated with immune cell infiltration (**Figures S2A–F**). According to the TCGA database, the SLFN5, SLFN12, SLFN12L, and SLFN14 expressions in GC were primarily related to T cell infiltration, particularly Tcm cell infiltration, whereas SLFN11 was primarily related to macrophages and dendritic cells (DCs) and SLFN13 was not significantly related to tumor immune cell infiltration (**Figures 7A–F**). The outcome is consistent with TIMER database. We used TISIDB database to examine the correlation between SLFN expression and immune cell infiltration and SLFN effect on the tumor microenvironment (TME). The findings were consistent, indicating that the SLFN5, SLFN11, and SLFN12 expression was positively correlated with natural killer (NK) cells, Th17 cells, and Treg cells in GC (**Figure S3**).

### SLFN expression correlates with immune checkpoint

Most members of SLFN family expression in GC were positively correlated with immune cell infiltration; earlier research demonstrated that tumor immune cell infiltration is associated with immune-related checkpoint expression (33). Based on this hypothesis, we further investigated whether SLFN expression in GC is related to immunological checkpoints. We investigated the relationship between SLFN5, SLFN11, SLFN12, SLFN13, CD160, CD244, CD247, CTLA4, LAG3, PDCD1, PDCD1LG2, TIGIT, and HAVCR2 using the TISIDB database. While SLFN12L and SLFN14 were excluded from the TISIDB database, we studied their link using GEPIA database. The results suggested that SLFN5, SLFN11, SLFN12, and SLFN12L were positively linked with CD160, CD244, CD247, CTLA4, LAG3, PDCD1, PDCD1LG2, TIGIT, and HAVCR2. However, there was no significant correlation between SLFN13 and these immunological checkpoints (**Figures S4A-D**). SLFN14 expression was positively correlated with CD160, CD244, CD247, PDCD1, PDCD1LG2, and TIGIT, but not with CTLA4, LAG3, and HAVCR2 (**Table 3** and **Figure S5**).

**Table 3:** Correlation between SLFN and immune checkpoint.

Immune checkpoint		SLFN5		SLFN11		SLFN12		SLFN12L		SLFN13		SLFN14	
		Cor	p	Cor	p	Cor	p	Cor	p	Cor	p	Cor	p
**CD160**		0.139	**	0.081	0.097	0.207	***	0.33	***	-0.029	0.551	0.33	***
**CD244**		0.37	***	0.329	***	0.298	***	0.62	***	-0.084	0.087	0.29	***
**CD247**		0.416	***	0.030	***	0.221	***	0.76	***	0.017	0.729	0.057	0.25
**CTLA4**		0.305	***	0.28	***	0.202	***	0.093	0.059	-0.008	0.864	0.034	0.49
**LAG3**		0.253	***	0.288	***	0.192	***	0.43	***	-0.036	0.466	0.059	0.24
**PDCD1**		0.314	***	0.317	***	0.174	***	0.51	***	-0.052	0.295	0.22	***
**PDCD1LG2**		0.5	***	0.487	***	0.366	***	0.52	***	-0.015	0.765	0.11	*
**TIGIT**		0.422	***	0.36	***	0.29	***	0.73	***	-0.018	0.719	0.35	***
**HAVCR2**		0.411	***	0.446	***	0.279	***	0.46	***	-0.039	0.432	0.093	0.061

*P<0.05, **P<0.01, ***P<0.001.

### Correlation between SLFN expression and immune cell markers

Our previous results revealed that increased SLFN5, SLFN11, SLFN12, SLFN12L, and SLFN14 expression in GC positively correlated with immune cell infiltration and immune checkpoints activation. We used the GEPIA database to verify the correlation between SLFN5, SLFN11, SLFN12, and SLFN12L expression and numerous immune cell features in GC. The results are listed in **Tables 3**, **4** as the gene set used to characterize immune cells. The immune cells included T cells, CD8^+^ T cells, B cells, M1 macrophages, M2 macrophages, monocytes, tumor-associated macrophages (TAMs), neutrophils, NK cells, and DCs. The findings revealed that the SLFN5, SLFN11, SLFN12, and SLFN12L expressions were strongly linked with most immunological markers in various types of immune cells (**Table 4**). As previous research indicates that the SLFN family is closely related to T cell activation, we further examined the correlation between SLFN and various T cells, including the effect of T cells, Treg, resting Treg, the effect of Treg, immature T cells, the effect of memory T cells, against memory T cells, and helper T cells Th1, Th1-like, and Th17 cells (**Table 5**).

**Table 4:** Correlation analysis between SLFN and gene markers of immune cells in GEPIA.

Description	Gene markers	SLFN5		SLFN11		SLFN12		SLFN12L	
		p	Cor	p	Cor	p	Cor	p	Cor
M2	CD163	***	0.34	***	0.38	***	0.34	***	0.33
	VSIG4	***	0.23	***	036	***	0.22	***	0.17
Neutrophils	MS4A4A	***	0.33	***	0.38	***	0.32	***	0.3
	CEACAM8	0.19	0.065	***	0.17	0.36	0.0046	0.8	-0.012
	ITGAM	***	0.37	***	0.3	***	0.3	***	0.37
Natural killer cell	CCR7	***	0.37	***	0.19	***	0.21	***	0.54
	KIR2DL1	*	0.13	**	0.13	**	0.13	***	0.24
	KIR2DL3	***	0.23	**	0.14	***	0.18	***	0.32
	KIR2DL4	***	0.23	0.15	0.0071	*	0.11	***	0.23
Dendritic	KIR3DL1	***	0.16	***	0.17	***	0.19	***	0.32
	KIR3DL2	***	0.35	***	0.21	***	0.25	***	0.45
	HLA-DPA1	***	0.28	***	0.28	***	0.28	***	0.47
cell	CD1C	***	0.27	***	0.21	***	0.23	***	0.43
	NRP1	***	0.47	***	0.37	***	0.32	***	0.3
	ITGAX	***	0.42	***	0.34	***	0.37	***	0.41
B cell	CD19	*	0.1	0.16	0.07	0.23	0.059	***	0.27
	CD79A	0.19	0.065	0.29	0.053	0.59	0.026	***	0.22
T cell	CD3D	***	0.28	***	0.19	***	0.26	***	0.61
	CD3E	***	0.39	***	0.24	***	0.35	***	0.72
	CD2	***	0.42	***	0.29	***	0.41	***	0.72
CD8+ T cell	CD8A	***	0.37	***	0.27	***	0.31	***	0.72
	CD8B	0.21	0.062	0.57	0.028	0.14	0.074	***	0.23
Monocyte	CD86	***	0.34	***	0.34	***	0.33	***	0.38
	CSF1R	***	0.42	***	0.41	***	0.36	***	0.4
TAM	CCL2	0.072	0.089	**	0.15	*	0.1	**	0.13
	CD68	***	0.24	***	0.25	***	0.18	**	0.16
	IL10	*	0.12	***	0.2	0.07	0.09	0.59	-0.027
M1	IRF5	***	0.32	***	0.21	***	0.19	***	0.28
	PTGS2	**	0.16	*	0.1	**	0.13	0.6	-0.026

*P<0.05, **P<0.01, ***P<0.001.

**Table 5:** Correlation between SLFN and gene markers of T cells in GEPIA.

Description	Gene markers	SLFN5		SLFN11		SLFN12		SLFN12L	
		p	Cor	p	Cor	p	Cor	p	Cor
Resident memory T-	CD69	***	0.37	***	0.29	***	0.32	***	0.55
cell	CXCR6	***	0.4	***	0.32	***	0.41	***	0.68
	MYADM	***	0.17	***	0.18	***	0.2	***	0.22
General	CCR7	***	0.37	***	0.19	***	0.21	***	0.54
memory T-cell	SELL	***	0.39	***	0.25	***	0.27	***	0.56
	IL7R	***	0.49	***	0.3	***	0.36	***	0.48
Exhausted T-cell	HAVCR2	***	0.42	***	0.38	***	0.32	***	0.46
	LAG3	***	0.24	*	0.12	***	0.22	***	0.43
	CXCL13	***	0.22	*	0.13	***	0.17	***	0.48
Th1	TBX21	***	0.4	***	0.28	***	0.39	***	0.77
	STAT4	***	0.48	***	0.3	***	0.44	***	0.74
	STAT1	***	0.44	***	0.34	***	0.42	***	0.45
	TNF	***	0.25	**	0.15	**	0.15	**	0.15
	IFNG	***	0.23	*	0.1	***	0.28	***	0.34
Th1-like	HAVCR2	***	0.42	***	0.38	***	0.32	***	0.46
	CXCR3	***	0.3	***	0.17	***	0.23	***	0.63
	BHLHE40	***	0.18	**	0.14	0.17	0.0069	0.48	-0.035
	CD4	***	0.44	***	0.39	***	0.4	***	0.59
Th2	STAT6	***	0.35	***	0.25	***	0.31	**	0.16
	STAT5A	***	0.56	***	0.37	***	0.42	***	0.51
Treg	FOXP3	***	0.39	***	0.24	***	0.33	***	0.58
	CCR8	***	0.48	***	0.28	***	0.35	***	0.6
	TGFB1	***	0.33	***	0.28	***	0.23	***	0.34
Resting Treg	FOXP3	***	0.39	***	0.24	***	0.33	***	0.58
	IL2RA	***	0.43	***	0.31	***	0.38	***	0.46
Effector Treg T-	FOXP3	***	0.39	***	0.24	***	0.33	***	0.58
cell	CCR8	***	0.48	***	0.28	***	0.35	***	0.6
	TNFRSF9	***	039	***	0.26	***	0.33	***	0.57
Effector T-cell	CX3CR1	***	0.21	*	0.11	***	0.21	***	0.3
	FGFBP2	0.058	0.094	0.33	0.049	*	0.12	0.17	0.068
	FCGR3A	***	0.34	***	0.32	***	0.32	***	0.37
Naive T-cell	CCR7	***	0.37	***	0.19	***	0.21	***	0.54
	SELL	***	0.39	***	0.25	***	0.27	***	0.56
Effector memory	DUSP4	0.88	-0.0076	0.11	-0.079	0.24	-0.059	*	-0.099
T-cell	GZMK	***	0.32	***	0.22	***	0.22	***	0.64
	GZMA	***	0.27	**	0.14	***	0.22	***	0.41

*p < 0.05, **p < 0.01, ***p < 0.001.

### The SLFN family expression in PBMC

**Figure 8:**
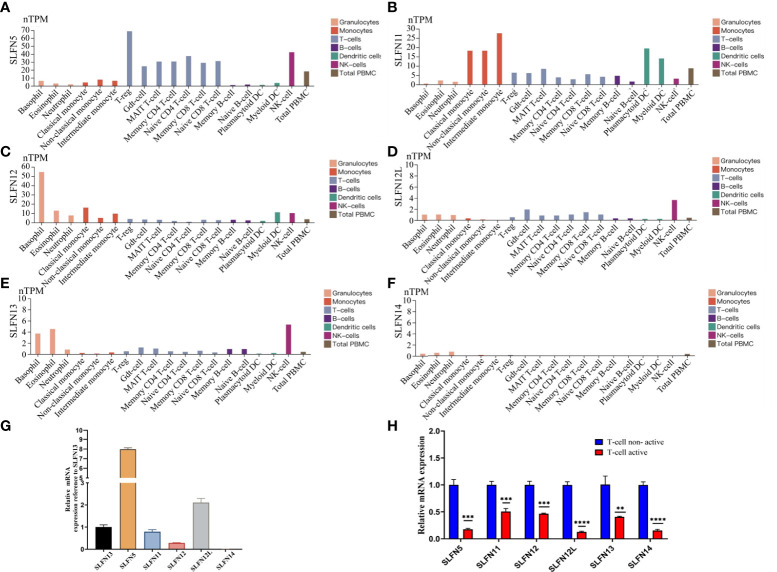
Expression of SLFN family in PBMC. **(A)** SLFN5 was highly expressed in Treg cells, NK cells, naive CD4 T cells, and naive CD8 T cells. **(B)** SLFN11 was highly expressed in monocytes and dendritic cells. **(C)** SLFN12 was highly expressed in basophils and monocytes. **(D)** SLFN12L was highly expressed in NK cells. **(E)** was highly expressed in eosinophils, basophils, and NK cells. **(F)** SLFN14 had generally low expression. **(G, H)** SLFN family mRNA expression was down-regulated after active of T cells. **(G)** In PBMC, compared with SLFN13, the expression level of SLFN5 was the highest, followed by SLFN12L, and the expression level of SLFN13 was the third. Compared with other families, the expression level of SLFN14 was negligible. **(H)** Changes of SLFN family mRNA expression before and after T cell activation. **p < 0.01 and ***p < 0.001.

According to our findings, SLFN family plays an important role in the immune microenvironment of GC and is functionally connected to various immune cell regulation. To further understand the role of SLFN family in immune cells, we used the Human Protein Atlas database that helps to study the SLFN family expression in PBMC. The results revealed that SLFN5 is expressed in Treg cells, NK cells, naive CD4^+^ T cells, and naive CD8^+^ T cells, SLFN11 is expressed in monocytes and DCs, SLFN12 is overexpressed in basophils and monocytes and SLFN12L is overexpressed in eosinophils, basophils and NK cells, SLFN13 is overexpressed in NK cells, while SLFN14 is expressed in NK cells (**Figures 8A-F**).

### Down-regulated SLFN expression in activation of T cells

The SLFN family expression in the PBMC indicates that SLFN5 is mainly expressed in Treg cells, which promote tumor progression (34). We hypothesize that elevated SLFN family expression in GC can regulate T cells resting state and induce GC progression. We examined the SLFN expression in inactivated and activated T cells to validate our hypothesis further. We isolated PBMC from healthy human peripheral blood. Cultured PBMC were amplified and then divided into two groups. One group received no special treatment, while the other was provided T cell activation reagent CD2/CD3/CD28 for 24 h before extracting RNA for performing RT-PCR. The results showed that SLFN5 had the greatest expression level in PBMC, followed by SLFN12L and SLFN13. SLFN14 expression was very low and insignificant compared to other members (**Figure 8G**). The SLFN5, SLFN11, SLFN12, SLFN12L, SLFN13, and SLFN14 expressions were significantly down-regulated after T cells activation, with SLFN12L being the most down-regulated, followed by SLFN5 and SLFN14 (**Figure 8H**). These findings imply that the SLFN family plays a crucial role in T cell activation and that its elevated expression in malignancies may inhibit the T cells activation.

## Discussion

The involvement of SLFN family members in cancer has recently received much attention. However, there are currently few studies on its role in GC (9). The role of SLFN in GC and whether SLFN family expression is linked to immune cell infiltration in GC remains unclear.

In this study, we systematically explored the expression, prognostic value, the biological function, the possible involved signaling pathways of the SLFN family in GC, and the role of SLFN family members in tumor-associated infiltrated immune cells. TCGA database revealed that six SLFN family members were highly expressed in various tumors. Except for SLFN14, the expression of the other family members was over-expressed in GC compared to normal gastric tissues in paired and unpaired analyses. The presence of the SLFN family was linked to tumor stage, lymph node metastasis, and tumor grade in GC. According to KM Plotter analysis, high SLFN5 and SLFN13 expression were associated with a poor prognosis, i.e., a lower OS and PFS. These findings imply that the SLFN family may enhance GC progression and that SLFN5 and SLFN13 can be employed as prognostic indicators for the disease. However, this result requires more experimental validation.

According to KEGG enrichment analysis, SLFN is involved in several inflammatory and infectious disorders, including malaria, hepatitis C, measles, EB virus infection, rheumatoid arthritis, and IBD. Furthermore, the signal pathways implicated are primarily associated with inflammation, such as the JAK-STAT signal and NF-κB signal pathway. The STRING database revealed that three possible target proteins interacted with SLFN, including BST2, SAMHD1, and TRIM5, previously implicated in viral infection (35–37). Inflammation plays a crucial role in tumor occurrence and growth, and some researchers argue that inflammation is the seventh characteristic of tumors (38, 39). A range of cytokines, including TNF, RANKL, IL-1, and IL-6, induce inflammation and promote tumor cell metastatic capacity through altering dissemination and colonization in distant metastatic lesions (40–42). The NF-κB signaling route can trigger inflammatory factors, and activate the NF-κB signaling pathway (40).

Tumor-associated immune cells can promote tumor progression by producing cytokines and assist tumor metastasis by secreting growth factors or matrix-degrading enzymes (43). Our study found that the SLFN5, SLFN11, SLFN12, SLFN12L, and SLFN14 expressions were positively linked with the infiltration of CD8^+^ T cells CD4^+^ T cells, macrophages, neutrophils, and dendritic cells. Furthermore, SLFN5, SLFN11, and SLFN12 were positively linked with NK cells, Th17 cells, and Treg cells in GC. Their expression levels were not significantly correlated to B cell infiltration and SLFN13 expression was not significantly correlated with immune cell infiltration. We predicted that some SLFN family members would enhance GC growth by increasing tumor immune cell infiltration.

In recent years, immune infiltrating cells in TME have been paid more attention for their crucial role in tumor genesis and development (44). Previous research has indicated that it contains tumor antagonistic or tumor-promoting properties (45). Tumor-associated immune cells are classified into two groups based on their function in tumors: tumor antagonistic immune cells and tumor-promoting immune cells (46). Tumor antagonistic immune cells are mainly composed of effector T cells (CD8^+^ T and CD4^+^ T cells), NK cells, DCs, M1-type macrophages, and N1-polarized neutrophils. Tumor-promoting immune cells are mainly composed of regulatory T cells (Treg) and myeloid suppressor cells (MDSCs) (45).

A previous study found SLFN5 co-localization with T cells and M2-type macrophages in precancerous lesions of GC, implying that SLFN5 plays an immunosuppressive role in GC (23). Consistently, we found that SLFN5 is primarily expressed in Treg and naïve T cells, which play a tumor-promoting role in cancer (47). Similarly, high SLFN11 expression in breast cancer is related to more aggressive and immune-activated tumors, whereas low SLFN11 expression is associated with low aggressiveness and low immune activation status (48). In a study of lung cancer recurrence, researchers discovered that the recurrence group had a higher density of Treg, M0, and M1 macrophage cells. In contrast, the density of memory B cells was higher in the control group. Further investigation revealed that Treg cells were substantially associated with lung cancer recurrence and that SLFN13 was one of the immune-related predictors of lung cancer recurrence (49). This research on SLFN family members promoting cancer appears to be related to another function of the family; i.e., they can regulate immune function and T cells to maintain a resting state (50).

Tumor antagonistic immune cells tend to destroy tumor cells in the early stages of tumor development (51). Still, tumor cells can eventually escape immune surveillance through various mechanisms, and even inhibit the cytotoxic effects of immune cells, according to research (51). One mechanism involved in immune escape is tumor cell expression at immune checkpoints, such as T cells expressing PD1, which bind to PD-L1 or PD-L2 ligands corresponding to the surface of tumor cells, causing T cells to become silent thus unable to kill tumors (52). The identified immune checkpoints include CTLA-4 (53), PD-1 (54), TIM-3 (55, 56), LAG-3 (57), TIGIT (58, 59), CD96, etc. (59). Currently, the therapies targeting immune checkpoints play an important role in tumor therapy. For example, CTLA-4 antibodies (Ipilimumab, Tremelimumab) block CTLA-4, and PD-1/PD-L1 antibodies (Nivolumab, Pembrolizumab, Durvalumab) block PD1. PD-1/PD-L1 and CTLA-4 antibodies effectively treat GC (60). Our study showed that the SLFN5, SLFN11, and SLFN12 expressions were positively correlated with CD160, CD244, CD247, CTLA4, LAG3, PDCD1, PDCD1LG2, TIGIT, and HAVCR2. SLFN14 expression was positively associated with CD160, CD244, CD247, PDCD1, PDCD1LG2, and TIGIT. This finding suggests that the SLFN family not only plays a role in immune cell infiltration of GC, but also participates in the expression of numerous immunological checkpoints. In conclusion, SLFN family not only predicts tumor immune cell infiltration but also serves as a predictor of immune checkpoint expression, which may play a role in therapeutic efficacy prediction or therapeutic target in gastric cancer immunotherapy. However, the conclusion requires further experimental verification.

We stimulated T cells in peripheral blood monocytes and demonstrated that the SLFN family’s expression was considerably downregulated. This result demonstrated that the SLFN family might play an important role in maintaining T cells in a non-activated state. Consistent with our results, a previous study has revealed that SLFN5 has high expression in T cells and decreases when T cells are activated, consistent with our findings. Furthermore, when INFa stimulated astrocytes and dendritic cells, SLFN5 expression increased. These findings suggested that SLFN5 may regulate T cells and other immune cells (13, 61). SLFN11 is found mostly in monocytes and dendritic cells, while SLFN12 is found primarily in monocytes, dendritic cells, and T cells. Both are down-regulated after differential stimulation (61). Several studies have consistently revealed that SLFN12 overexpression in T cells can maintain the quiet state of T cells (62).

As homologous of the human SLFN family, SLFN1 and SLFN2 in mice are the main SLFN family proteins that regulate the resting state of T cells, and SLFN1 maintains the resting state of T cells by blocking T cell cycle progression from cytoplasm to nucleus [27]. In mice, SLFN2 -/- mouse led to T cells over-activation [28], whereas SLFN3 is predominantly produced in CD4^+^ CD25^+^ Treg cells but down-regulated after T cell activation [29].

In conclusion, this study investigated the role and potential molecular mechanism of the SLFN family in GC. The SLFN5 and SLFN13 expressions in GC may play a significant role in cancer promotion and can be utilized to predict the GC prognosis. Additionally, it was inferred that SLFN family expression is favorably associated with immune cell infiltration and immunological checkpoints in GC. After T cells are activated, the SLFN family is drastically downregulated, and earlier research has shown that SLFN can help regulatory T cells maintain their quiet state. Based on the above opinion, we hypothesize that increased SLFN family expression results from tumor immune cell infiltration and immune checkpoints activation. Notably, it can induce T cells into a quiescent state, resulting in immunological escape in GC, although additional experimental evidence is required to confirm our hypothesis.

## Conclusion

SLFN5 and LFN13 are prognostic markers for GC, and SLFN family members SLFN5, SLFN11, SLFN12, SLFN12L, and SLFN14 are associated with immune cell infiltration and immune checkpoint expression in GC, which may promote GC progression by inhibiting T cell activation and thus mediating immune escape in GC.

## Data availability statement

The data showed in this article can be found in online repositories. The detail of repositories can be seen in Materials and Methods of this article and **Supplementary Material**

## Ethics statement

This study was reviewed and approved by Medical Ethics Committee of the Seventh Affiliated Hospital of Sun Yat-Sen University. The patients/participants provided their written informed consent to participate in this study.

## Author contributions

Study concept and design: JX, CZ and YH. Acquisition of data: JX, SC, JL, and TH. Analysis and interpretation of data: CZ, YH, JX, SC, JL, TH, GL, HW and XJ. Statistical analysis: JX, SC, JL, HL and JZ. Drafting of the manuscript: JX SC and JL. Critical revision and final approval of the manuscript: JX, CZ and YH. Obtained funding: CZ and YH. Study supervision: YH. All authors contributed to the article and approved the submitted version.

## Funding

This study was supported by Guangdong Provincial Key Laboratory of Digestive Cancer Research (No. 2021B1212040006), Sanming Project of Medicine in Shenzhen (No. SZSM201911010), Shenzhen Key Medical Discipline Construction Fund (No. SZXK016) and Shenzhen Sustainable Project (KCXFZ202002011010593).

## Conflict of interest

The authors declare that the research was conducted in the absence of any commercial or financial relationships that could be construed as a potential conflict of interest.

## Publisher’s note

All claims expressed in this article are solely those of the authors and do not necessarily represent those of their affiliated organizations, or those of the publisher, the editors and the reviewers. Any product that may be evaluated in this article, or claim that may be made by its manufacturer, is not guaranteed or endorsed by the publisher.

## Supplementary material

Supplementary Figure 1 GO Analysis (Biological process) of SLFN family in GC. **(A-F)** Biological process of SLFN family in GC cohort.

Supplementary Figure 2 Tumor immune cell infiltration associated to SLFN. **(A-F)** The relationship between the expression of SLFN family members and tumor immune cells in gastric cancer was analyzed using Timer database with purity-corrected partial Spearman correlation test. **(A-D, F)** The expression of SLFN5, SLFN11, SLFN12, and SLFN12L in gastric cancer was significantly positively correlated with the infiltration of CD8^+^ T cells, CD4^+^ T cells, macrophages, neutrophils, and dendritic cells, but not with the absence of infiltration of B cells. **(E)** SLFN13 expression in gastric cancer has no significant relationship with immune cell infiltration. **(F)** SLFN14 is positively correlated with all infiltration immune cells.

Supplementary Figure 3 Tumor immune cell infiltration associated to SLFN. Tumor immune cell infiltration associated to SLFN in CRC investigated by TISIDB database. **(A-C)** The expression of SLFN5, SLFN11, and SLFN12 was positively correlated with NK cells, Th17 cells and Treg cells in gastric cancer.

Supplementary Figure 4 Relationship between SLFN family members and immune checkpoints. TISIDB database was used to analyze the correlation between SLFN5, SLFN11, SLFN12, and SLFN13, and CD160, CD244, CD247, CTLA4, LAG3, PDCD1, PDCD1LG2, TIGIT, and HAVCR2. **(A-D)** SLFN5, SLFN11, and SLFN12 were positively correlated with CD160, CD244, CD247, CTLA4, LAG3, PDCD1, PDCD1LG2, TIGIT, and HAVCR2 in gastric cancer. **(D)** SLFN13 expression in gastric cancer has no significant correlation with immune checkpoint.

Supplementary Figure 5 Relationship between SLFN family members and immune checkpoints. **(A)**SLFN12L were positively linked with CD160, CD244, CD247, CTLA4, LAG3, PDCD1, PDCD1LG2, TIGIT, and HAVCR2. **(B)** SLFN14 expression was positively correlated with CD160, CD244, CD247, PDCD1, PDCD1LG2, and TIGIT, but not with CTLA4, LAG3, and HAVCR2

## References

[1] Allemani C Matsuda T Di Carlo V Harewood R Matz M Nikšić M. Global surveillance of trends in cancer survival 2000-14 (CONCORD-3): analysis of individual records for 37 513 025 patients diagnosed with one of 18 cancers from 322 population-based registries in 71 countries. Lancet (2018) 391(10125):1023–75. doi: 10.1016/s0140-6736(17)33326-3 PMC5879496 29395269

[2] Sung H Ferlay J Siegel RL Laversanne M Soerjomataram I Jemal A. Global cancer statistics 2020: GLOBOCAN estimates of incidence and mortality worldwide for 36 cancers in 185 countries. CA Cancer J Clin (2021) 71(3):209–49. doi: 10.3322/caac.21660 33538338

[3] Rugge M Capelle LG Cappellesso R Nitti D Kuipers EJ. Precancerous lesions in the stomach: from biology to clinical patient management. Best Pract Res Clin Gastroenterol (2013) 27(2):205–23. doi: 10.1016/j.bpg.2012.12.007 23809241

[4] Zong L Abe M Seto Y Ji J. The challenge of screening for early gastric cancer in China. Lancet (2016) 388(10060):2606. doi: 10.1016/s0140-6736(16)32226-7 27894662

[5] Patel SP Kurzrock R. PD-L1 expression as a predictive biomarker in cancer immunotherapy. Mol Cancer Ther (2015) 14(4):847–56. doi: 10.1158/1535-7163.Mct-14-0983 25695955

[6] Herbst RS Soria JC Kowanetz M Fine GD Hamid O Gordon MS. Predictive correlates of response to the anti-PD-L1 antibody MPDL3280A in cancer patients. Nature (2014) 515(7528):563–7. doi: 10.1038/nature14011 PMC4836193 25428504

[7] Schwarz DA Katayama CD Hedrick SM. Schlafen, a new family of growth regulatory genes that affect thymocyte development. Immunity (1998) 9(5):657–68. doi: 10.1016/s1074-7613(00)80663-9 9846487

[8] Liu F Zhou P Wang Q Zhang M Li D. The schlafen family: complex roles in different cell types and virus replication. Cell Biol Int (2018) 42(1):2–8. doi: 10.1002/cbin.10778 28460425

[9] Al-Marsoummi S Vomhof-Dekrey EE Basson MD. Schlafens: Emerging proteins in cancer cell biology. Cells (2021) 10(9):2238. doi: 10.3390/cells10092238 34571887 PMC8465726

[10] Katsoulidis E Mavrommatis E Woodard J Shields MA Sassano A Carayol N. Role of interferon {alpha} (IFN{alpha})-inducible schlafen-5 in regulation of anchorage-independent growth and invasion of malignant melanoma cells. J Biol Chem (2010) 285(51):40333–41. doi: 10.1074/jbc.M110.151076 PMC3001013 20956525

[11] Sassano A Mavrommatis E Arslan AD Kroczynska B Beauchamp EM Khuon S. Human schlafen 5 (SLFN5) is a regulator of motility and invasiveness of renal cell carcinoma cells. Mol Cell Biol (2015) 35(15):2684–98. doi: 10.1128/mcb.00019-15 PMC4524119 26012550

[12] Wan G Zhu J Gu X Yang Y Liu Y Wang Z. Human schlafen 5 regulates reversible epithelial and mesenchymal transitions in breast cancer by suppression of ZEB1 transcription. Br J Cancer (2020) 123(4):633–43. doi: 10.1038/s41416-020-0873-z PMC7435190 32488136

[13] Arslan AD Sassano A Saleiro D Lisowski P Kosciuczuk EM Fischietti M. Human SLFN5 is a transcriptional co-repressor of STAT1-mediated interferon responses and promotes the malignant phenotype in glioblastoma. Oncogene (2017) 36(43):6006–19. doi: 10.1038/onc.2017.205 PMC5821504 28671669

[14] Fischietti M Eckerdt F Blyth GT Arslan AD Mati WM Oku CV. Schlafen 5 as a novel therapeutic target in pancreatic ductal adenocarcinoma. Oncogene (2021) 40(18):3273–86. doi: 10.1038/s41388-021-01761-1 PMC8106654 33846574

[15] Martinez RS Salji MJ Rushworth L Ntala C Rodriguez Blanco G Hedley A. SLFN5 regulates LAT1-mediated mTOR activation in castration-resistant prostate cancer. Cancer Res (2021) 81(13):3664–78. doi: 10.1158/0008-5472.Can-20-3694 33985973

[16] Zoppoli G Regairaz M Leo E Reinhold WC Varma S Ballestrero A. Putative DNA/RNA helicase schlafen-11 (SLFN11) sensitizes cancer cells to DNA-damaging agents. Proc Natl Acad Sci U S A (2012) 109(37):15030–5. doi: 10.1073/pnas.1205943109 PMC3443151 22927417

[17] Tian L Song S Liu X Wang Y Xu X Hu Y. Schlafen-11 sensitizes colorectal carcinoma cells to irinotecan. Anticancer Drugs (2014) 25(10):1175–81. doi: 10.1097/cad.0000000000000151 25089570

[18] Nogales V Reinhold WC Varma S Martinez-Cardus A Moutinho C Moran S. Epigenetic inactivation of the putative DNA/RNA helicase SLFN11 in human cancer confers resistance to platinum drugs. Oncotarget (2016) 7(3):3084–97. doi: 10.18632/oncotarget.6413 PMC4823092 26625211

[19] De Waal L Lewis TA Rees MG Tsherniak A Wu X Choi PS. Identification of cancer-cytotoxic modulators of PDE3A by predictive chemogenomics. Nat Chem Biol (2016) 12(2):102–8. doi: 10.1038/nchembio.1984 PMC4718766 26656089

[20] Ding L Hayes MM Photenhauer A Eaton KA Li Q Ocadiz-Ruiz R. Schlafen 4-expressing myeloid-derived suppressor cells are induced during murine gastric metaplasia. J Clin Invest (2016) 126(8):2867–80. doi: 10.1172/jci82529 PMC4966326 27427984

[21] Merchant JL Ding L. Hedgehog signaling links chronic inflammation to gastric cancer precursor lesions. Cell Mol Gastroenterol Hepatol (2017) 3(2):201–10. doi: 10.1016/j.jcmgh.2017.01.004 PMC5331830 28275687

[22] El-Zaatari M Kao JY Tessier A Bai L Hayes MM Fontaine C. Gli1 deletion prevents helicobacter-induced gastric metaplasia and expansion of myeloid cell subsets. PloS One (2013) 8(3):e58935. doi: 10.1371/journal.pone.0058935 23520544 PMC3592845

[23] Companioni Nápoles O Tsao AC Sanz-Anquela JM Sala N Bonet C Pardo ML. SCHLAFEN 5 expression correlates with intestinal metaplasia that progresses to gastric cancer. J Gastroenterol (2017) 52(1):39–49. doi: 10.1007/s00535-016-1202-4 27032393 PMC5045746

[24] Chandrashekar DS Bashel B Balasubramanya S a H Creighton CJ Ponce-Rodriguez I Chakravarthi B. UALCAN: A portal for facilitating tumor subgroup gene expression and survival analyses. Neoplasia (2017) 19(8):649–58. doi: 10.1016/j.neo.2017.05.002 PMC5516091 28732212

[25] Gao J Aksoy BA Dogrusoz U Dresdner G Gross B Sumer SO. Integrative analysis of complex cancer genomics and clinical profiles using the cBioPortal. Sci Signal (2013) 6(269):pl1. doi: 10.1126/scisignal.2004088 23550210 PMC4160307

[26] Warde-Farley D Donaldson SL Comes O Zuberi K Badrawi R Chao P. The GeneMANIA prediction server: biological network integration for gene prioritization and predicting gene function. Nucleic Acids Res (2010) 38(Web Server issue):W214–20. doi: 10.1093/nar/gkq537 PMC2896186 20576703

[27] Vasaikar SV Straub P Wang J Zhang B. LinkedOmics: analyzing multi-omics data within and across 32 cancer types. Nucleic Acids Res (2018) 46(D1):D956–d63. doi: 10.1093/nar/gkx1090 PMC5753188 29136207

[28] Lánczky A Győrffy B. Web-based survival analysis tool tailored for medical research (KMplot): Development and implementation. J Med Internet Res (2021) 23(7):e27633. doi: 10.2196/27633 34309564 PMC8367126

[29] Li T Fu J Zeng Z Cohen D Li J Chen Q. TIMER2.0 for analysis of tumor-infiltrating immune cells. Nucleic Acids Res (2020) 48(W1):W509–w14. doi: 10.1093/nar/gkaa407 PMC7319575 32442275

[30] Ru B Wong CN Tong Y Zhong JY Zhong SSW Wu WC. TISIDB: an integrated repository portal for tumor-immune system interactions. Bioinformatics (2019) 35(20):4200–02. doi: 10.1093/bioinformatics/btz210 30903160

[31] Tang Z Li C Kang B Gao G Li C Zhang Z. GEPIA: a web server for cancer and normal gene expression profiling and interactive analyses. Nucleic Acids Res (2017) 45(W1):W98–w102. doi: 10.1093/nar/gkx247 28407145 PMC5570223

[32] Uhlen M Karlsson MJ Zhong W Tebani A Pou C Mikes J. A genome-wide transcriptomic analysis of protein-coding genes in human blood cells. Science (2019) 366(6472):eaxx9198. doi: 10.1126/science.aax9198 31857451

[33] Teng MW Ngiow SF Ribas A Smyth MJ. Classifying cancers based on T-cell infiltration and PD-L1. Cancer Res (2015) 75(11):2139–45. doi: 10.1158/0008-5472.Can-15-0255 PMC4452411 25977340

[34] Liang J Bi G Shan G Jin X Bian Y Wang Q. Tumor-associated regulatory T cells in non-Small-Cell lung cancer: Current advances and future perspectives. J Immunol Res (2022) 2022:4355386. doi: 10.1155/2022/4355386 35497874 PMC9054468

[35] Chen S Huang X Xie Q Liu Q Zhu H. The role of long noncoding RNA BST2-2 in the innate immune response to viral infection. J Virol (2022) 96(7):e0020722. doi: 10.1128/jvi.00207-22 35297670 PMC9006898

[36] Resop RS Bosque A. Pharmacological targeting of sphingosine kinases impedes HIV-1 infection of CD4 T cells through SAMHD1 modulation. J Virol (2022) 96(9):e0009622. doi: 10.1128/jvi.00096-22 35412343 PMC9093127

[37] Désaulniers K Ortiz L Dufour C Claudel A Plourde MB Merindol N. Editing of the TRIM5 gene decreases the permissiveness of human T lymphocytic cells to HIV-1. Viruses (2020) 13(1):24. doi: 10.3390/v13010024 PMC7824555 33375604

[38] Mantovani A. Cancer: Inflaming metastasis. Nature (2009) 457(7225):36–7. doi: 10.1038/457036b 19122629

[39] Greten FR Grivennikov SI. Inflammation and cancer: Triggers, mechanisms, and consequences. Immunity (2019) 51(1):27–41. doi: 10.1016/j.immuni.2019.06.025 31315034 PMC6831096

[40] Mantovani A Allavena P Sica A Balkwill F. Cancer-related inflammation. Nature (2008) 454(7203):436–44. doi: 10.1038/nature07205 18650914

[41] Giavazzi R Garofalo A Bani MR Abbate M Ghezzi P Boraschi D. Interleukin 1-induced augmentation of experimental metastases from a human melanoma in nude mice. Cancer Res (1990) 50(15):4771–5. 2196116

[42] Kondoh N Mizuno-Kamiya M. The role of immune modulatory cytokines in the tumor microenvironments of head and neck squamous cell carcinomas. Cancers (Basel) (2022) 14(12):2884. doi: 10.3390/cancers14122884 35740551 PMC9221278

[43] Bi Q Wu JY Qiu XM Zhang JD Sun ZJ Wang W. Tumor-associated inflammation: The tumor-promoting immunity in the early stages of tumorigenesis. J Immunol Res (2022) 2022:3128933. doi: 10.1155/2022/3128933 35733919 PMC9208911

[44] Binnewies M Roberts EW Kersten K Chan V Fearon DF Merad M. Understanding the tumor immune microenvironment (TIME) for effective therapy. Nat Med (2018) 24(5):541–50. doi: 10.1038/s41591-018-0014-x PMC5998822 29686425

[45] Lei X Lei Y Li JK Du WX Li RG Yang J. Immune cells within the tumor microenvironment: Biological functions and roles in cancer immunotherapy. Cancer Lett (2020) 470:126–33. doi: 10.1016/j.canlet.2019.11.009 31730903

[46] Pansy K Uhl B Krstic J Szmyra M Fechter K Santiso A. Immune regulatory processes of the tumor microenvironment under malignant conditions. Int J Mol Sci (2021) 22(24):13311. doi: 10.3390/ijms222413311 34948104 PMC8706102

[47] Wang J Gong R Zhao C Lei K Sun X Ren H. Human FOXP3 and tumor microenvironment. Immunology (2022). doi: 10.1111/imm.13520 35689826

[48] Isnaldi E Ferraioli D Ferrando L Brohée S Ferrando F Fregatti P. Schlafen-11 expression is associated with immune signatures and basal-like phenotype in breast cancer. Breast Cancer Res Treat (2019) 177(2):335–43. doi: 10.1007/s10549-019-05313-w 31222709

[49] Wang Q Zhou D Wu F Liang Q He Q Peng M. Immune microenvironment signatures as biomarkers to predict early recurrence of stage ia-b lung cancer. Front Oncol (2021) 11:680287. doi: 10.3389/fonc.2021.680287 34395248 PMC8356052

[50] De La Casa-Esperón E. From mammals to viruses: the schlafen genes in developmental, proliferative and immune processes. Biomol Concepts (2011) 2(3):159–69. doi: 10.1515/bmc.2011.018 25962026

[51] Hanahan D Weinberg RA. Hallmarks of cancer: the next generation. Cell (2011) 144(5):646–74. doi: 10.1016/j.cell.2011.02.013 21376230

[52] Munari E Mariotti FR Quatrini L Bertoglio P Tumino N Vacca P. PD-1/PD-L1 in cancer: Pathophysiological, diagnostic and therapeutic aspects. Int J Mol Sci (2021) 22(10):5123. doi: 10.3390/ijms22105123 34066087 PMC8151504

[53] Leach DR Krummel MF Allison JP. Enhancement of antitumor immunity by CTLA-4 blockade. Science (1996) 271(5256):1734–6. doi: 10.1126/science.271.5256.1734 8596936

[54] Freeman GJ Long AJ Iwai Y Bourque K Chernova T Nishimura H. Engagement of the PD-1 immunoinhibitory receptor by a novel B7 family member leads to negative regulation of lymphocyte activation. J Exp Med (2000) 192(7):1027–34. doi: 10.1084/jem.192.7.1027 PMC2193311 11015443

[55] Sánchez-Fueyo A Tian J Picarella D Domenig C Zheng XX Sabatos CA. Tim-3 inhibits T helper type 1-mediated auto- and alloimmune responses and promotes immunological tolerance. Nat Immunol (2003) 4(11):1093–101. doi: 10.1038/ni987 14556005

[56] Das M Zhu C Kuchroo VK. Tim-3 and its role in regulating anti-tumor immunity. Immunol Rev (2017) 276(1):97–111. doi: 10.1111/imr.12520 28258697 PMC5512889

[57] Lichtenegger FS Rothe M Schnorfeil FM Deiser K Krupka C Augsberger C. Targeting LAG-3 and PD-1 to enhance T cell activation by antigen-presenting cells. Front Immunol (2018) 9:385. doi: 10.3389/fimmu.2018.00385 29535740 PMC5835137

[58] Joller N Kuchroo VK. Tim-3, lag-3, and TIGIT. Curr Top Microbiol Immunol (2017) 410:127–56. doi: 10.1007/82_2017_62 PMC5902028 28900677

[59] Dougall WC Kurtulus S Smyth MJ Anderson AC. TIGIT and CD96: new checkpoint receptor targets for cancer immunotherapy. Immunol Rev (2017) 276(1):112–20. doi: 10.1111/imr.12518 28258695

[60] Yoneda A Kuroki T Eguchi S. Immunotherapeutic advances in gastric cancer. Surg Today (2021) 51(11):1727–35. doi: 10.1007/s00595-021-02236-2 33590326

[61] Puck A Aigner R Modak M Cejka P Blaas D Stöckl J. Expression and regulation of schlafen (SLFN) family members in primary human monocytes, monocyte-derived dendritic cells and T cells. Results Immunol (2015) 5:23–32. doi: 10.1016/j.rinim.2015.10.001 26623250 PMC4625362

[62] Puck A Hopf S Modak M Majdic O Cejka P Blüml S. The soluble cytoplasmic tail of CD45 (ct-CD45) in human plasma contributes to keep T cells in a quiescent state. Eur J Immunol (2017) 47(1):193–205. doi: 10.1002/eji.201646405 27718235 PMC5244668

