# Supplementary material for: Schlafen family is a prognostic biomarker and corresponds with immune infiltration in gastric cancer
Source: Front Immunol. 2022 Aug 25;13:922138. doi: 10.3389/fimmu.2022.922138 (PMC9452737; doi:10.3389/fimmu.2022.922138)
Supplement: Supplementary file 1 [file Table_1.pdf]

Table S1 The primers used in the methods

Primers	Forward	Reverse
SLFN5	ACAGACGTGTCACACTGTGTT	CCCTCAAGCTCGTAAGGTCTAT
SLFN11	CAGCCTGACAACCGAGAAATG	GGCCCACTAGATAGACTCAGC
SLFN12	TTGGAAACGAATTATGCCGAGT	AGAGCACACATAGCTCGTGAG
SLFN12L	CCCAGAGTTATCCTCTTCGTGA	CCCTTATTGACAGAGCCCATTTC
SLFN13	CTCTTCAAAACCTCGGGTAGAGT	CCACACAACAGAATGCCTTCA
SLFN14	CAGCCTTCCACTAAGGATTTGC	GCTCTAAACCCCTTCTCTCTGAG
