# Supplementary figures and images for: Schlafen family is a prognostic biomarker and corresponds with immune infiltration in gastric cancer
Source: Front Immunol. 2022 Aug 25;13:922138. doi: 10.3389/fimmu.2022.922138 (PMC9452737; doi:10.3389/fimmu.2022.922138)

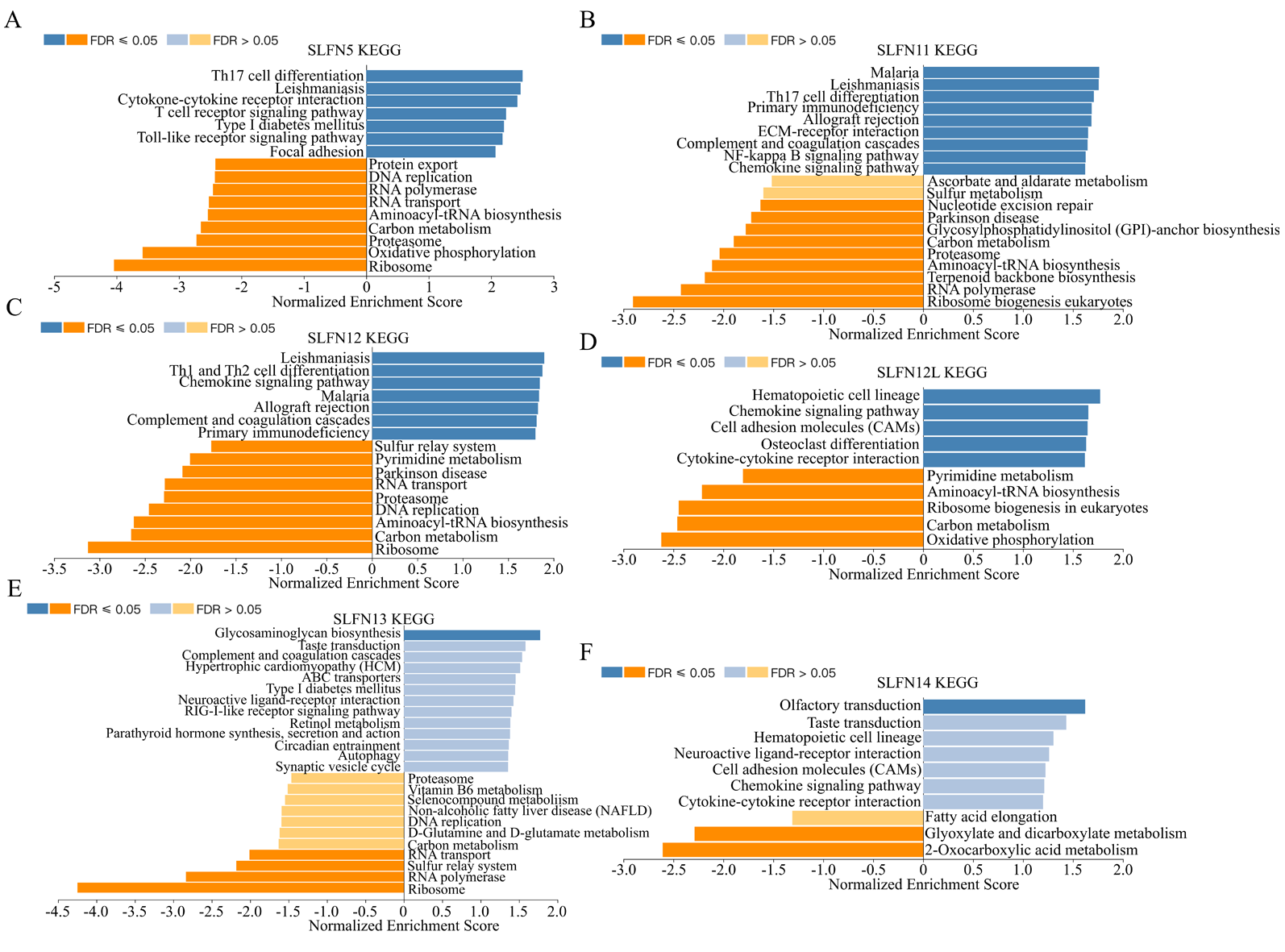

Supplement: Supplementary Figure 1 — GO Analysis (Biological process) of SLFN family in GC. (A-F) Biological process of SLFN family in GC cohort. [file Image_1.tif]

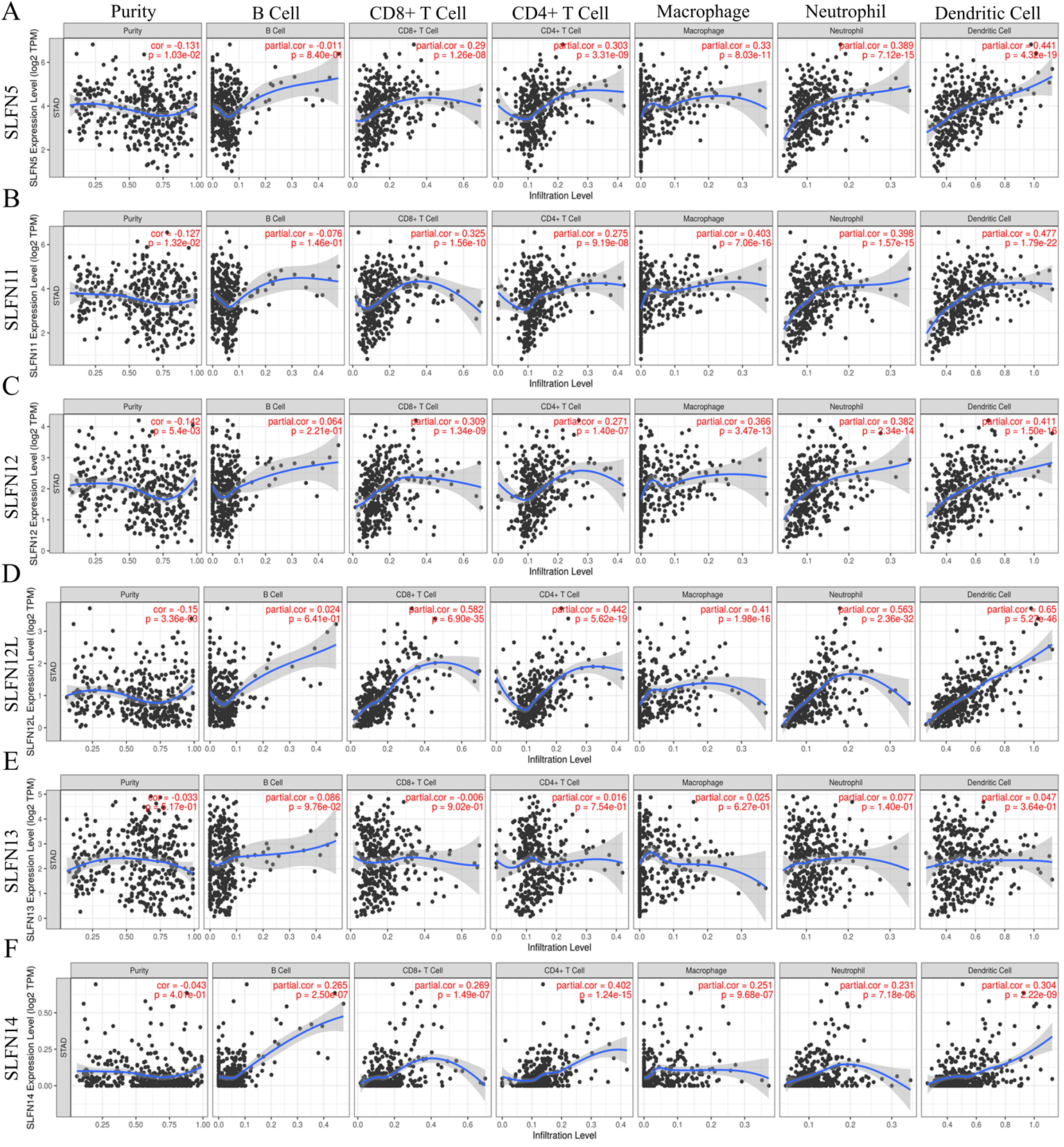

Supplement: Supplementary Figure 2 — Tumor immune cell infiltration associated to SLFN. (A-F) The relationship between the expression of SLFN family members and tumor immune cells in gastric cancer was analyzed using Timer database with purity-corrected partial Spearman correlation test. (A-D, F) The expression of SLFN5, SLFN11, SLFN12, and SLFN12L in gastric cancer was significantly positively correlated with the infiltration of CD8+ T cells, CD4+ T cells, macrophages, neutrophils, and dendritic cells, but not with the absence of infiltration of B cells. (E) SLFN13 expression in gastric cancer has no significant relationship with immune cell infiltration. (F) SLFN14 is positively correlated with all infiltration immune cells. [file Image_2.tif]

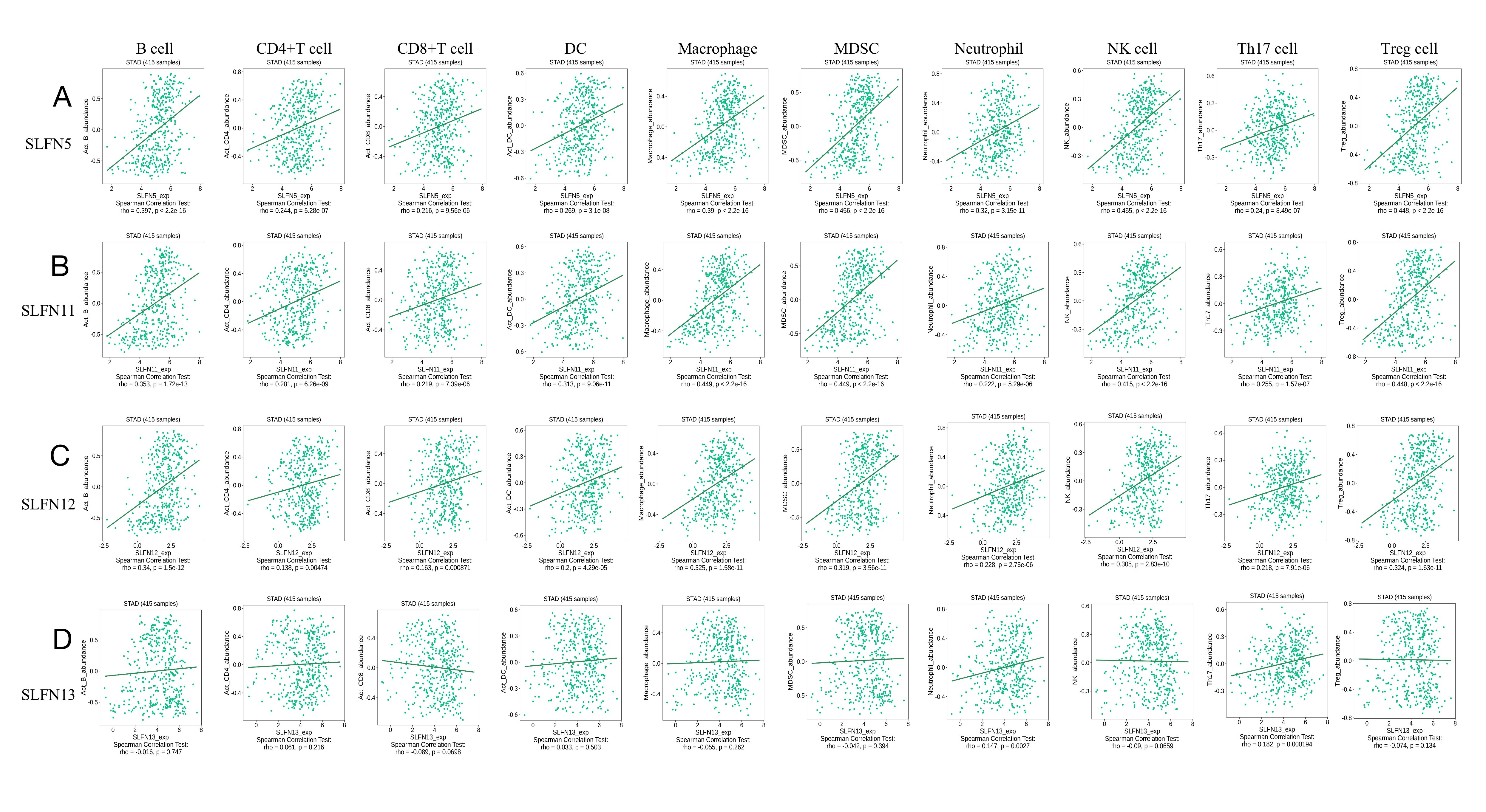

Supplement: Supplementary Figure 3 — Tumor immune cell infiltration associated to SLFN. Tumor immune cell infiltration associated to SLFN in CRC investigated by TISIDB database. (A-C) The expression of SLFN5, SLFN11, and SLFN12 was positively correlated with NK cells, Th17 cells and Treg cells in gastric cancer. [file Image_3.tif]

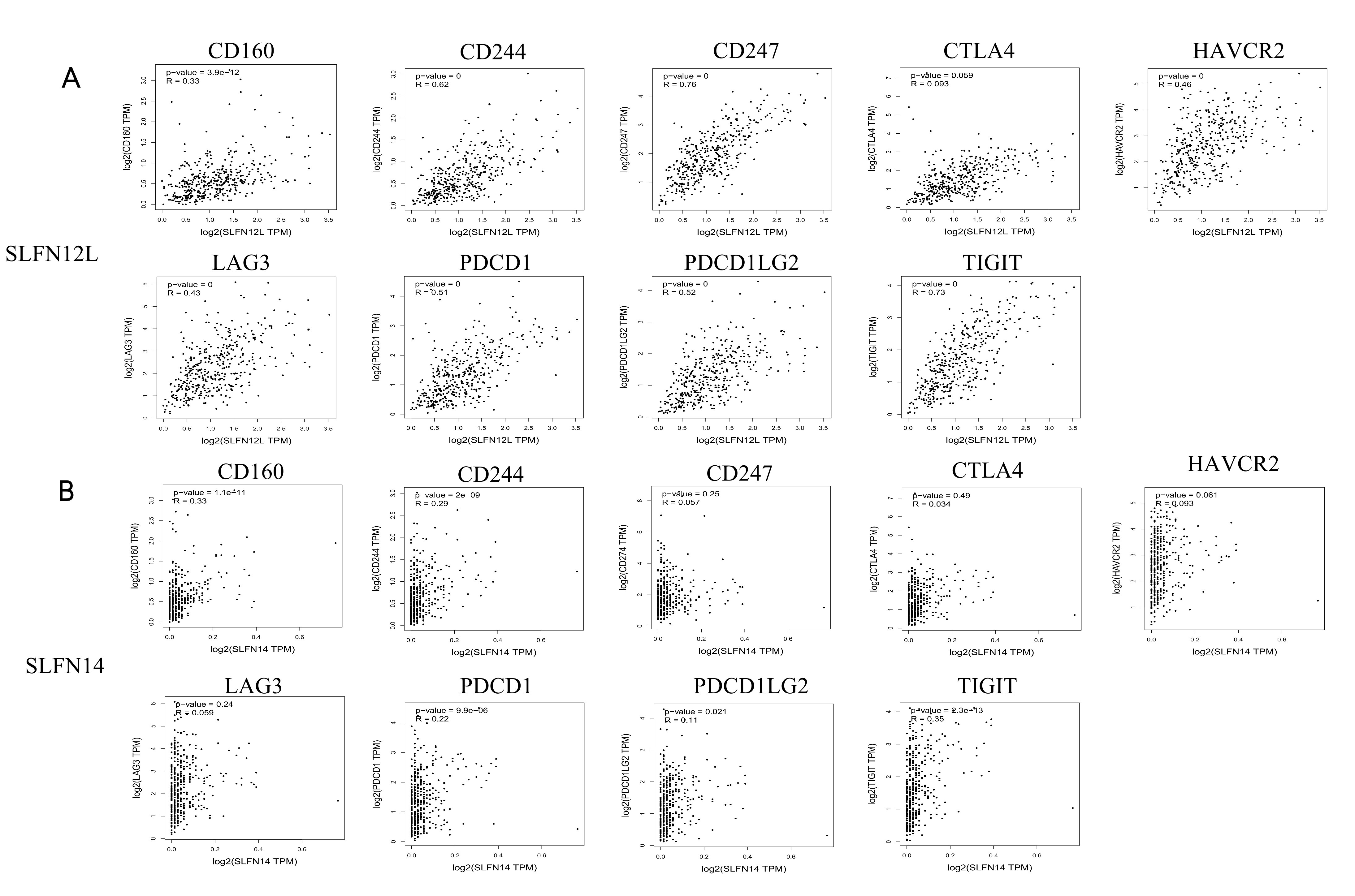

Supplement: Supplementary Figure 5 — Relationship between SLFN family members and immune checkpoints. (A)SLFN12L were positively linked with CD160, CD244, CD247, CTLA4, LAG3, PDCD1, PDCD1LG2, TIGIT, and HAVCR2. (B) SLFN14 expression was positively correlated with CD160, CD244, CD247, PDCD1, PDCD1LG2, and TIGIT, but not with CTLA4, LAG3, and HAVCR2 [file Image_5.tif]
